# Genotypic Variation and Genetic Control of Phenolic Compounds and Antioxidant Activity in Shanlan Upland Rice Landrace

**DOI:** 10.3390/ijms26199800

**Published:** 2025-10-08

**Authors:** Lin Zhang, Jing Yu, Bowen Deng, Yi Peng, Yafang Shao, Jinsong Bao

**Affiliations:** 1 Hainan Institute, Zhejiang University, Yazhou Bay Science and Technology City, Yazhou District, Sanya 572025, China; 2 Institute of Nuclear Agricultural Sciences, Key Laboratory of Nuclear Agricultural Sciences of Ministry of Agriculture and Rural Affairs, College of Agriculture and Biotechnology, Zhejiang University, Zijingang Campus, Hangzhou 310058, China; 3 China National Rice Research Institute, Hangzhou 310006, China

**Keywords:** Shanlan rice, phenolic compounds, genome-wide association study

## Abstract

Shanlan rice, a unique drought-resistant rice germplasm resource in Hainan Province, China, holds significant potential for rice genetic improvement and breeding innovation. However, its genetic diversity and significance in rice breeding remain inadequately explored. This study conducted a comprehensive analysis of phenolic acid profiles and antioxidant properties in the brown rice of 84 Shanlan rice accessions. It was revealed that colored Shanlan rice accessions exhibited significantly higher total phenolic content (249.00–2408.33 mg gallic acid equivalents per 100 g of rice flour (mg GAE/100 g)) and antioxidant capacity (DPPH: 680.39–809.63 micromoles of Trolox equivalent per 100 g (μmol TE/100 g); ABTS: 529.93–1917.77 μmol TE/100 g) compared to white-grained varieties. High-performance liquid chromatography (HPLC) analysis identified eight phenolic acids in the bound fractions, among which the sinapic acid (55.08 μg/g) and vanillic acid (11.72 μg/g) were predominant, accounting for over 60% of total bound phenolic acid content. A genome-wide association study (GWAS) identified 84 significant loci associated with these phenolic-related traits. A major quantitative trait locus (QTL) on chromosome 7 for free phenolic content, total phenolic content, flavonoids, and DPPH activity was co-located at the *Rc* gene locus, a key regulator of red pericarp pigmentation and proanthocyanidin biosynthesis. Haplotype analysis identified ten haplotypes in *Rc*, with the haplotype H002 showing the highest antioxidant capacity. Another QTL on chromosome 11 was associated with p-coumaric, vanillic, and sinapic acids, although no significant difference was observed in haplotype analysis. These results highlight *Rc* as a key genetic factor underlying antioxidant properties in rice, while other loci require further validation. This research provides a foundation for breeding health-benefit, drought-tolerant rice cultivars using Hainan’s unique germplasm.

## 1. Introduction

Rice *(Oryza sativa* L.) is the primary caloric source for over 3.5 billion people, contributing > 20% of global dietary energy intake [1]. Brown rice, produced by dehusking without subsequent milling, retains its nutrient-dense bran layers, which are rich in phenolic acids, γ-oryzanols, dietary fiber, and tocotrienols. Accumulating epidemiological evidence suggests that higher intake of whole-grain brown rice is associated with a reduced risk of type 2 diabetes and cardiovascular disease [2,3,4,5]. While these associations are likely influenced by overall dietary and lifestyle patterns, components such as dietary fiber and phenolic compounds have been proposed as potential contributors to these health benefits based on their bioactive properties. In particular, rice bran phenolics have demonstrated antioxidant, anti-inflammatory, and enzyme-inhibitory activities in preclinical models [6]. Plant phenolics are broadly classified based on chemical structure into flavonoids—characterized by a C6–C3–C6 skeleton—and non-flavonoid phenolics. The latter include phenolic acids (e.g., hydroxybenzoic and hydroxycinnamic acid derivatives), simple phenols, coumarins, stilbenes, tannins (hydrolyzable and condensed), lignans, and lignin. These compounds are primarily synthesized through the phenylpropanoid metabolic pathway, which originates from phenylalanine produced via the shikimate pathway [7].

Previous studies have identified the presence of protocatechuic acid, p-hydroxybenzoic acid, vanillic acid, syringic acid, ferulic acid, and sinapic acid in whole grains of rice [8,9,10]. Ferulic acid is frequently reported as the predominant phenolic compound in brown rice, particularly in the bound fraction of rice bran [11,12]. However, due to variations in genetic background and extraction/hydrolysis methods, the relative abundance of phenolic acids has been shown to vary significantly across studies. Generally, colored rice (such as red, purple, and black rice) has higher total phenolic and flavonoid content and antioxidant capacity than white rice, as demonstrated in multiple studies [13,14]. Therefore, the color parameters of rice are positively correlated with the content of total phenolics, flavonoids, and antioxidant capacity [15].

Genetic analysis indicated that many regulatory genes are involved in flavonoid biosynthesis, which significantly influences grain pigmentation in rice [16,17]. Shao et al. reported significant associations between two rice SSR markers, RM339 (chromosome 8) and RM316 (chromosome 9), and the accumulation of phenolic compounds, flavonoids, and antioxidant capacity [18]. The *Ra* gene (also known as *Prp-b*) and the *Rc* locus have been established as major determinants of both grain coloration and nutritional quality traits [18]. Using a whole-genome association study (GWAS), Xu et al. [19] identified two key genomic regions associated with total flavonoid and polyphenol content: *Os06g0611725* on chromosome 6 and *Os07g0211500* on chromosome 7. Within the white rice subgroup, a distinct locus for total polyphenol content was detected at *Os10g0372100* on chromosome 10 [19]. SSR marker-based association analyses by Li et al. identified additional genetic factors, with Rid12 (chromosome 7), RM484 (chromosome 10), RM162 (chromosome 6), and RM5371 (chromosome 6) showing significant associations with phenolic content, flavonoid accumulation, and antioxidant capacity, respectively [20]. More recently, multi-environment GWAS using 96 diverse rice germplasm accessions identified genomic regions associated with total flavonoid and polyphenol content [21]. The analyses revealed AX-95920592 (chromosome 2) and AX-95926544 (chromosome 5) as significant genetic loci regulating total polyphenol accumulation in whole rice grains [21].

Shanlan landrace rice, as a unique local variety of upland rice in Hainan Province, China, not only has distinct germplasm characteristics and favorable agronomic traits but also exhibits remarkable ecological adaptability. It is recognized as a special germplasm resource in rice breeding and possesses extremely important social, ecological, economic, and cultivation values. In recent years, the increasing drought occurrences and the growing adoption of ecological farming practices have renewed interest in Shanlan rice as an exceptional upland rice variety. Consequently, its cultivation has gradually been reintroduced. Current research on Shanlan rice primarily focuses on drought resistance mechanisms [22,23] and starch properties [24]. However, its health-promoting traits, particularly phenolic compound content, remain understudied.

The objectives of this study are: (1) To investigate the genotypic diversity in the phenolic content, flavonoid content and bound phenolic acids in Shanlan rice accessions using high-performance liquid chromatography (HPLC); (2) To identify genomic loci associated with phenolics and antioxidant activity variation through GWAS; and (3) To propose candidate genes regulating phenolic metabolism by functional annotation of GWAS hits. The results obtained will provide valuable insights for the nutritional quality breeding of Shanlan landrace rice.

## 2. Results

### 2.1. Analysis of Phenolics and Flavonoids Content

**Figure 1:**
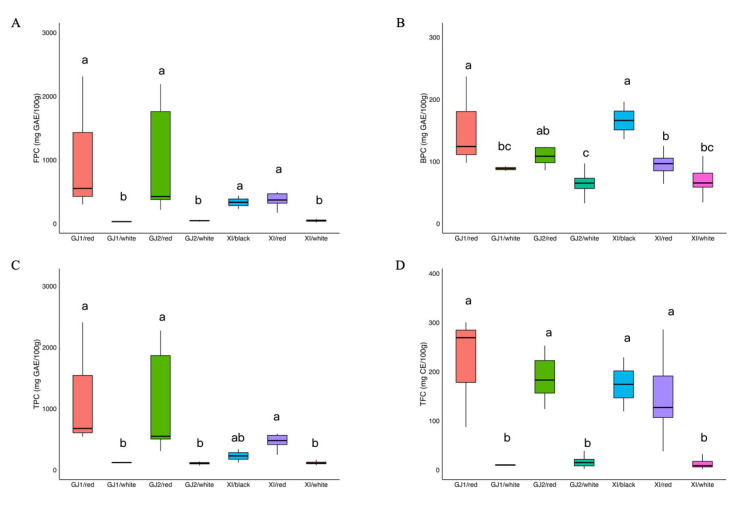
Levels of phenolic and flavonoid compounds in colored and white rice across different genetic groups. (**A**) Free phenolic content (FPC, mg GAE/100 g); (**B**) Bound phenolic content (BPC, mg GAE/100 g); (**C**) Total phenolic content (TPC, mg GAE/100 g); (**D**) Total flavonoid content (TFC, mg CE/100 g). Columns topped with different letters within the same genetic group indicate a statistically significant difference at *p* < 0.05. GJ1: Geng/Japonica 1; GJ2: Geng/Japonica 2; XI: Xian/Indica.

The contents of phenolics and flavonoids in Shanlan rice are shown in Figure 1. Previous studies have shown that all the rice accessions could be divided into three main groups, i.e., Geng/japonica 1 (GJ1), Geng/japonica 2 (GJ2), and Xian/indica (XI) [24] (Table S1). Red rice had an average free phenolic content (FPC) of 1055.4, 927.4, and 555.6 mg GAE/100 g in the GJ1, GJ2, and XI groups, which was markedly higher than that in white rice, which had an average of 31.9, 54.1, and 59.7 mg GAE/100 g in the GJ1, GJ2, and XI groups. Black rice in the XI group had an average FPC of 340.2 mg GAE/100 g (Figure 1A). Similarly, the bound phenolic content (BPC) followed a similar trend. The average BPC values for red rice across GJ1 and GJ2 groups were 153.0 and 105.1 mg GAE/100 g, respectively, significantly exceeding those of corresponding white rice (88.7 and 62.0 mg GAE/100 g). In the XI group, the average BPC value of red rice (97.1 mg GAE/100 g) did not differ significantly from that of the corresponding white rice (70.1 mg GAE/100 g) (*p* > 0.05) (Figure 1B). However, the average BPC value of black rice was 165.4 mg GAE/100 g, showing a significant difference from that of the corresponding white rice (Figure 1B). Furthermore, the colored rice varieties consistently showed significantly higher total phenolic content (TPC) and total flavonoid content (TFC) than white rice varieties (Figure 1C; Figure 1D).

### 2.2. Antioxidant Properties

Two methods, the DPPH and ABTS radical scavenging, were used to determine the antioxidant activities. As shown in Table 1, the colored rice, particularly red rice, exhibited significantly higher antioxidant capacity than the white rice. Red rice demonstrated DPPH radical scavenging activity ranging from 680.39 to 809.63 μmol TE/100 g, and ABTS radical cation scavenging activity between 529.93 and 1917.77 μmol TE/100 g.

### 2.3. Correlation Analysis

Pearson’s correlation analysis was performed to examine the relationships among FPC, BPC, TPC, TFC, and antioxidant activities (DPPH and ABTS) (Table 2). The results showed significant positive correlations between phenolic and flavonoid contents and both DPPH and ABTS values (all *p* < 0.01). In addition, TPC was strongly and positively correlated with TFC (*p* < 0.01), indicating that higher phenolic content was closely associated with increased flavonoid levels and enhanced antioxidant capacity.

### 2.4. Quantitative Analysis of Bound Phenolic Acids Compositions

High-performance liquid chromatography (HPLC) was used to identify the individual phenolic acids in the bound fractions. Table 1 presents the content of individual phenolic acids in bound fractions of Shanlan rice grains.

The results indicated no statistically significant differences in the levels of the eight identified phenolic acids among white, red, and black rice (Table 1). However, a closer look at the data revealed slight numerical variations in phenolic acid content between pigmented (red and black rice) and non-pigmented (white rice) varieties. Notably, gallic acid (GA), vanillic acid (VA), and sinapic acid (SA) tended to be present at higher concentrations in red and black rice compared to white rice. In contrast, protocatechuic acid (PA), p-hydroxybenzoic acid (p-HBA), caffeic acid (CA), p-coumaric acid (p-CA), and ferulic acid (FA) exhibited similar levels across all three rice types. These findings suggested that while certain phenolic acids might be more abundant in pigmented rice, others were uniformly distributed regardless of grain color. Further investigation may provide deeper insights into the specific contributions of these compounds to the nutritional profiles and potential health benefits of colored versus white rice. Notably, SA and VA were the most abundant phenolic acids across all samples, indicating their potential role in the antioxidant properties and pigment-related traits of Shanlan rice grains.

A correlation analysis was conducted between individual phenolic acids and BPC, as shown in Table 2. The results revealed significant positive correlations between GA and PA, as well as between GA and p-HBA (*p* < 0.01). Moreover, PA demonstrated a significant positive correlation with p-HBA. CA was significantly correlated with SA and FA. VA displayed significant correlations with p-CA, FA, and SA.

### 2.5. GWAS Analysis

**Figure 2:**
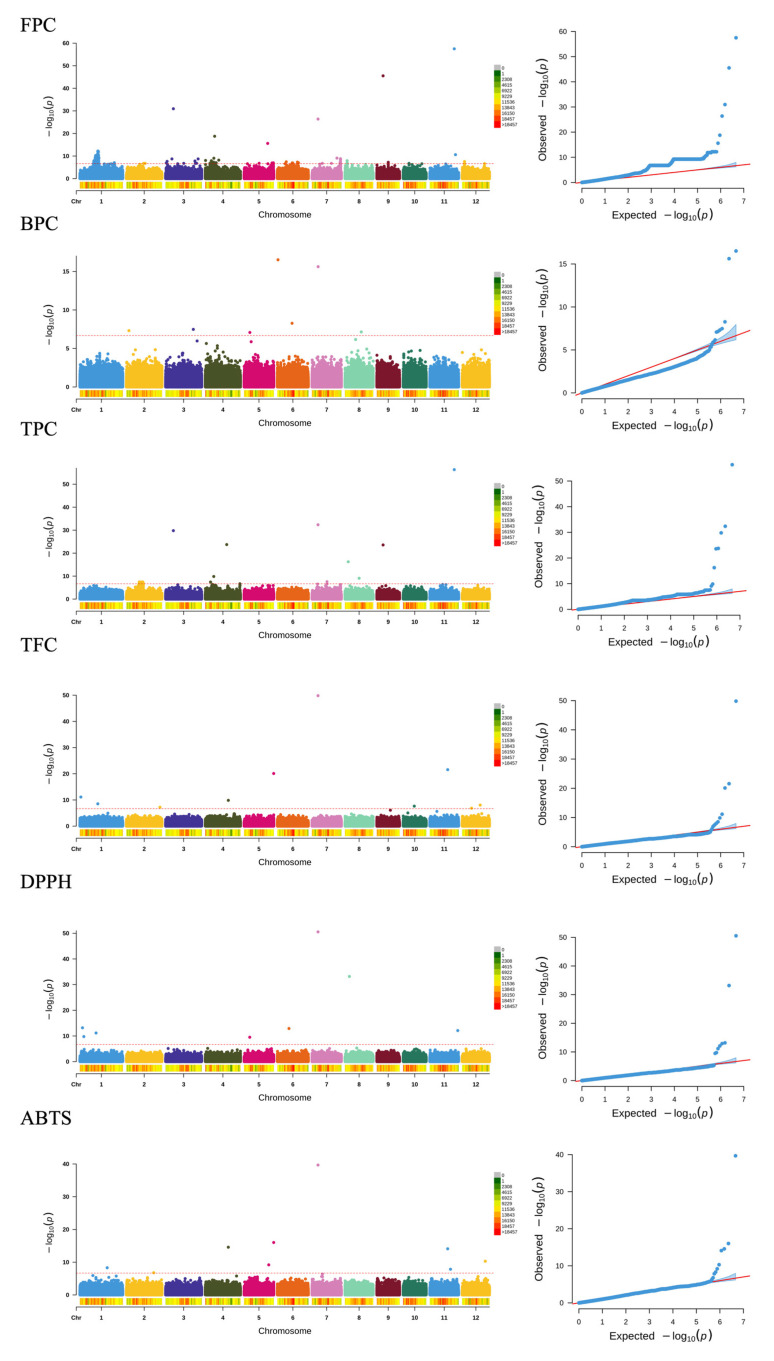

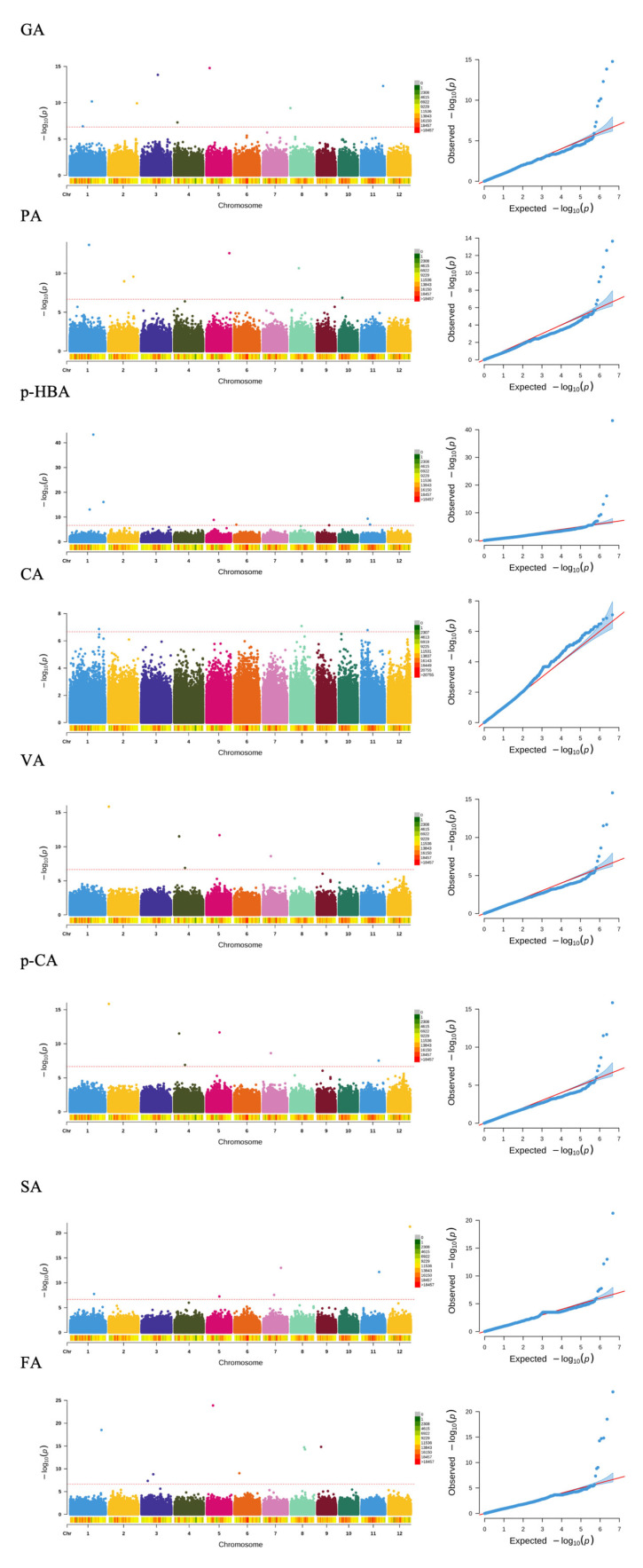
Manhattan plot (**left**) and QQ plot (**right**) of genome-wide association studies using the FarmCPU model on phenolic acid-related traits. The points in the Manhattan plots indicate the −log_10_(P) values. The horizontal red line indicates the significant thresholds.

A genome-wide association study utilizing 3,425,822 SNPs and insertion/deletion (InDel) markers revealed 84 significant loci linked to phenolic compounds, flavonoids, and antioxidant capacity (Figure 2). Linkage disequilibrium (LD) analysis showed that the physical distance corresponding to an *r^2^* threshold of 0.31 was 183 kb [24]. Based on this LD decay parameter, we defined a 183 kb genomic interval surrounding each significantly associated SNP as the candidate region. All genes within these candidate regions were extracted from the rice genome annotation database and subjected to further functional analysis (Table 3).

Notably, seven loci on chromosomes 4, 5, 6, 7, 8, and 12 were identified for FPC, with S7_6068071 displaying the most robust association signal across the population (*p* = 4.13 × 10^−27^). For total phenolic content (TPC), nine loci were identified on chromosomes 3, 4, 7, 8, 9, and 11, with S3_7796717, S7_6068071, and S11_24663643 showing notable association signals. Importantly, the identification of a shared locus between FPC and TPC suggested a strong correlation between these two traits. Ten loci associated with TFC were identified on chromosomes 1, 2, 4, 5, 7, 10, 11, and 12, with a notable association observed at locus S7_6068071. Eight loci on chromosomes 1, 5, 6, 7, 8, and 11 were detected for DPPH, with the strongest associations at loci S7_6068071 and S8_4407898. Nine loci on chromosomes 1, 2, 4, 5, 7, 11, and 12 were detected for ABTS, with the strongest association locus at locus S7_6068071, suggesting a potential regulatory role in antioxidant activity. Seven loci were detected for GA, with locus S5_3991938 displaying a strong association. Six loci were identified for PA, with locus S1_23469338 on chromosome 1 showing a strong association. Eight loci were identified for p-HBA, with a notable association observed on chromosome 1. Six loci were detected for SA, with the strongest association observed at locus S12_26390982. Eight loci were revealed for FA, with locus S5_7616250 showing the strongest association within the population. Interestingly, p-CA and VA shared six loci, with locus S2_551933 displaying a strong association.

### 2.6. Identification of Candidate Gene and Haplotype Analysis

**Figure 3:**
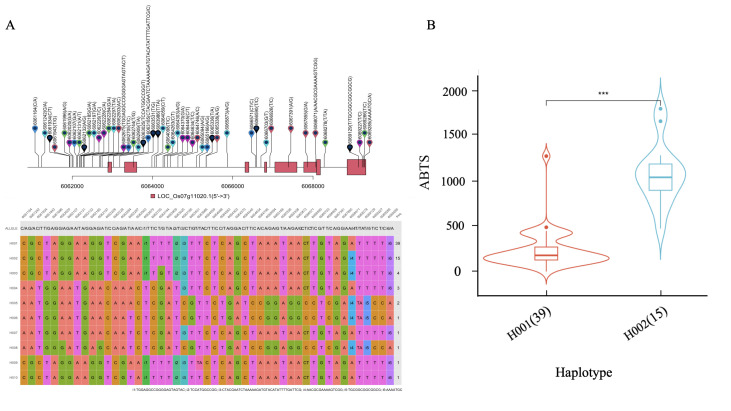
Identification of candidate genes for the whole panel. (**A**) Based on 19 SNPs in all evaluated rice accessions, 10 haplotypes of *Rc* (LOC_Os07g11020) were identified. In the gene structure diagram of LOC_Os07g11020 (http://rice.plantbiology.msu.edu, accessed on 1 September 2024.), the exon and untranslated regions are indicated by a red frame, and the intron and intergenic regions are marked by black lines. (**B**) ABTS radical scavenging capacity was compared among haplotype groups by Duncan’s multiple comparison test. Haplotypes with fewer than five accessions were excluded from the analysis. ***, *p* < 0.001.

A prominent peak for FPC on chromosome 7 coincided with peaks for TPC, TFC, DPPH scavenging activity, and ABTS scavenging activity (Figure 2), indicating a potential pleiotropic effect of a single gene on these crucial traits. Subsequent gene annotation efforts pinpointed this significant locus within the exon region of *Rc*, a gene encoding a protein featuring a basic helix-loop-helix (bHLH) motif known for regulating seed coat color and flavonoid biosynthesis [25]. Analysis of 19 SNPs in the *Rc* promoter, 5 SNPs in exonic regions, 21 SNPs in introns, and 4 SNPs in the 3′-untranslated region (3′-UTR) revealed ten distinct haplotypes across all studied accessions (Figure 3A). Notably, haplotype H002 exhibited enhanced levels of phenolic compounds and the highest antioxidant activity (Figure 3B).

**Figure 4:**
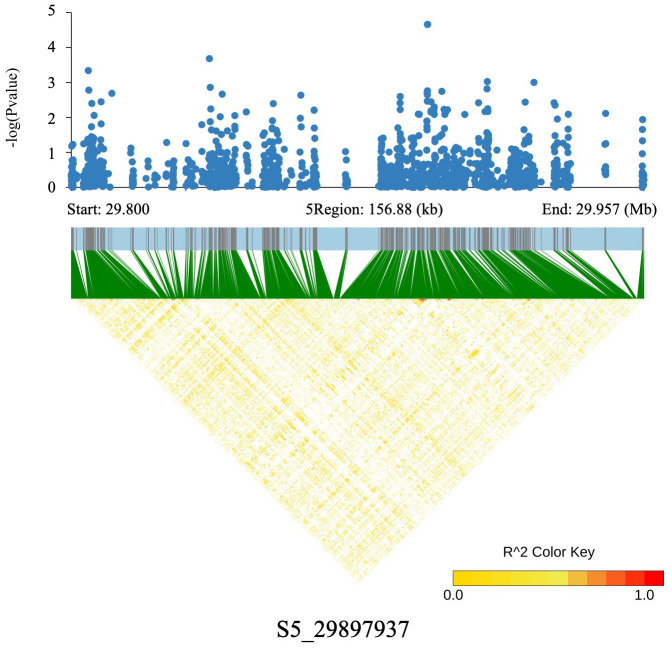
High-density gene-based association analysis and linkage disequilibrium heat map of the local Manhattan map around the peak on chromosome 5.

Association analysis revealed that TFC and ABTS radical scavenging activity shared a significant locus, S5_29897937, on chromosome 5, and a co-localized locus, S11_18026280, was identified on chromosome 11. For the S5_29897937 locus on chromosome 5, linkage disequilibrium (LD) analysis across the ~100 kb flanking genomic region showed no strong LD signals among genetic markers (low LD), suggesting that this locus may represent an independent genetic effect. However, the possibility of a false-positive association could not be ruled out (Figure 4). Further genomic annotation indicated that S5_29897937 is located within the exon of LOC_Os05g52070, a gene annotated as encoding a retrotransposon-derived transcriptional regulator, providing preliminary clues for its functional analysis.

**Figure 5:**
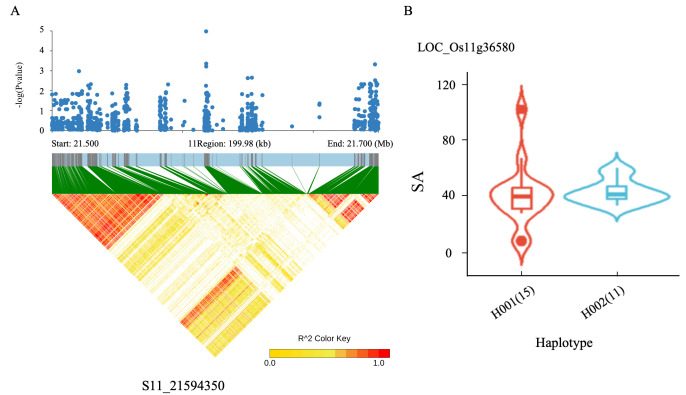
Identification of candidate genes for SA, p-CA, and VA. (**A**) High-density gene-based association analysis and linkage disequilibrium heat map of the local Manhattan map around the peak on chromosome 11. (**B**) SA comparisons among accessions carrying different haplotypes of LOC_Os11g36580. Haplotypes with fewer than five accessions were excluded from the analysis.

The SNP S11_21594350 on chromosome 11 was found to be a shared locus for p-CA, VA, and SA, located upstream of the gene LOC_Os11g36580. Haplotype analysis of this genomic region revealed no substantial variations among different haplotypes (Figure 5).

## 3. Discussion

### 3.1. Extensive Diversity in Phenolics of Shanlan Rice

This study investigated the total phenolic content, antioxidant characteristics, and specific phenolic acids in Shanlan rice, and analyzed the differences among different sub-populations and between different seed coat color groups. The results showed that across different groups, the free phenolic content was higher than the bound phenolic content, consistent with previous studies [26,27]. Zhang et al. [28] also found that the FPC was higher than the BPC in the white rice and black rice hybrid breeding lines. However, Pang et al. [29] pointed out that the content of insoluble bound phenolics was significantly higher than free phenolics. Actually, the relative abundance of free versus bound phenolics can vary significantly depending on extraction methodology, matrix properties, and genetic background. Free phenolics are easily extracted with polar solvents like 80% methanol [30], whereas bound phenolics require chemical treatments (e.g., alkaline or acid hydrolysis) to cleave covalent bonds with cell wall components, such as arabinoxylans [31]. Incomplete hydrolysis or oxidative degradation during extraction may lead to underestimation of BPC, potentially explaining discrepancies across studies. Furthermore, cultivar-specific differences in cell wall architecture and crosslinking density can influence the proportion of phenolics retained in the bound form [32]. Notably, although BPC may be less abundant in extracts, they exhibit delayed release in the colon via microbial fermentation, enhancing their bioaccessibility and potential health benefits in the lower gastrointestinal tract [33].

Both free and bound phenolics contents in colored rice varieties were significantly higher than those in white rice, consistent with previous studies [34,35]. The results of Min et al. [26] showed that the FPC of red rice and purple rice was significantly higher than that of white rice and other cereals, which may be due to the high concentration of anthocyanins in purple rice and procyanidins in red rice. In addition, research has found that there were no significant differences in the contents of 8 phenolic acid monomers among groups (Table 1). Sinapic acid and vanillic acid were identified as the dominant bound phenolic acids in this study, which contrasts with previous reports indicating ferulic acid, p-coumaric acid, and vanillic acid as the major phenolic acids in rice [36,37]. These discrepancies may be attributed to differences in rice types (e.g., colored vs. white rice), the chemical forms of phenolic acids analyzed (bound vs. free), and the methodological differences applied to their extraction.

Previous studies have demonstrated that phenolic acids, flavonoids, and antioxidant activity have significant correlations [15,20]. The results of this study also showed that the TPC and TFC were positively correlated with DPPH and ABTS scavenging ability (*r* = 0.742, *p* < 0.01). The significant correlation between p-coumaric acid, ferulic acid, and sinapic acid found by Xu et al. [19] was also verified in this study.

### 3.2. Genetic Basis of Phenolic Compounds and Antioxidant Activities in Rice

Research has shown that the formation of red rice grains is due to the presence of proanthocyanidins in the pericarp, a process regulated by the *Rc* and *Rd* genes [38]. When both *Rc* and *Rd* are functional, the pericarp is red [38]. When only *Rc* is functional, the pericarp is brown, and when only *Rd* is functional, the pericarp is white [38]. A genome-wide association study carried out by Xu et al. [19] established a correlation between the whole-grain color (red and white) and the antioxidant capacity along with phenolic components (both free and bound), thereby corroborating the participation of the *Rc* in all free phenolics and antioxidant activity. Similarly, Purnama et al. [39] evaluated the antioxidant properties of 233 Thai rice varieties using various methods, including ABTS, DPPH, iron reducing antioxidant capacity (FRAP), TFC, and TPC, and associated them with SNPs to identify regions important for antioxidant properties. The *Rc* gene was identified and consistently correlated with all phenotypic traits. In this study, *Rc* was unequivocally identified as a predominant determinant governing the total phenolic acid content, total flavonoid content, as well as the antioxidant activity within rice (Table 3), which is in consonance with previous literature [20,40]. In order to further understand the effect of the *Rc* genotype on rice quality, a comprehensive haplotype analysis was performed. This analysis led to the discovery of the superior haplotype H002, characterized by a significantly high phenolic acid content and strong antioxidant properties. 

For TPC, a significant SNP, namely S8_3232230, was precisely located in the vicinity of *pds1* (*panicle exsertion defect and aberrant spikelet*) on chromosome 8. The *pds1* is known to have an impact on the normal development of spikelets, resulting in exposed or abnormal spikelets [41]. Although our primary focus was on genetic variation influencing phenolic acid metabolism, this finding raises the possibility of a potential genetic or regulatory link between phenolic content and panicle development. Notably, previous studies have indicated that flavonoid compounds, which are closely related to phenolic metabolism, tend to accumulate during panicle development, with certain compounds such as hesperidin, delphinidin, and quercetin reaching peak levels at the heading stage [42]. These observations suggest that phenolic metabolism may be functionally or developmentally coordinated with inflorescence formation in rice. While the exact mechanism remains to be elucidated, the genomic region surrounding *pds1* may represent a hotspot for interactions between metabolic regulation and reproductive development. Further functional characterization of this locus could provide insights into how secondary metabolite biosynthesis and spikelet morphogenesis are genetically integrated. It is worth noting that this potential connection requires subsequent functional verification.

A TFC-associated SNP, S4_23251954, was identified near *OsGASR2*, a gibberellin-responsive gene involved in cell division and panicle differentiation [43]. This finding suggests a potential connection between phenolic acid metabolism and hormone-regulated developmental processes. Studies have shown that flavonoids can modulate plant hormone signaling pathways, particularly those related to auxin and ABA [44]. Given that phenolic acids serve as precursors or intermediates in flavonoid biosynthesis, our findings imply that genetic variation affecting phenolic acid content may indirectly influence hormonal homeostasis and developmental regulation in rice.

In addition, a significant SNP associated with GA-related traits (S8_166588) was localized near *OsVQ32*, a gene that functions as a substrate in the OsMPKK6–OsMPK4 signaling cascade and plays a positive role in rice resistance to bacterial blight [45]. Ferulic acid, a key phenolic compound derived from the phenylpropanoid pathway, has been shown to enhance plant tolerance to various abiotic stresses, including heat and salinity, and to modulate stress-responsive signaling pathways such as the mitogen-activated protein kinase (MAPK) cascade [46]. Notably, FA can interact with plant hormones such as abscisic acid (ABA) and ethylene, influencing the activity of transcription factors like *WRKY* and *MYB*, which are central regulators of stress-responsive gene expression [47]. Given that *OsVQ32* is involved in *MAPK* signaling and disease resistance, the proximity of a GA-associated SNP to this genomic region suggests a potential genetic or regulatory link between phenolic acid metabolism and plant stress adaptation. Together, these findings highlight the roles of phenolic acids in stress responses in rice.

For p-HBA, S1_41031628 was identified near *OsNF-YB6/OsHAP3G*. Heterotrimeric heme activator protein (HAP) complex is also known as CCAAT box factor (CBF) or nuclear factor Y (NF-Y). The HAP complex consists of three subunits: HAP2 (NF-YA; CBF-B), HAP3 (NF-YB; CBF-A), and HAP5 (NF-YC; CBF-C). This complex binds to the CCAAT sequence in the promoter to control the expression of target genes [48,49]. NF-Y is a ubiquitous transcription factor that regulates important physiological and developmental processes. Previous studies have identified 34 *OsNF-Y* genes in rice. *OsHAP3A* (*NF-YB2*), *OsHAP3B* (*NF-YB3*), and *OsHAP3C* (*NF-YB4*) have been shown to regulate chloroplast biosynthesis in rice [50]. Overexpression of *OsHAP3E* (*NF-YB7*) leads to various abnormal morphologies during both vegetative and reproductive stages [51]. *Ghd8* (*NF-YB11*) has multiple effects on rice yield, heading stage, and plant height [52]. *OsNF-YB1* is an endosperm-specific gene involved in maintaining the proliferation of endosperm cells [53]. *OsHAP2E* (*NF-YA2*) enhances resistance to biotic and abiotic stress and increases photosynthesis and tiller number [54]. *OsHAP5A*, *OsHAP5B*, *OsHAP3D*, and *OsHAP3E* delayed the heading stage under long-day conditions [49]. However, there have been no reports on the functional research of *OsNF-YB6*. This discovery implies it is necessary to investigate whether the function of *OsNF-YB6* is involved in phenolic acid biosynthesis.

Regarding p-CA and VA, S2_551933 was found near *DHD4*, which encodes a CONSTANS transcription factor that forms homodimers, influencing the formation of the florigen activation complex and leading to delayed flowering [55]. For FA, S3_7652662 is located near *OsAUX2*, which is an auxin efflux transporter protein also expressed in seeds, indicating that ferulic acid is involved in auxin transport. Previous studies have shown that phenolic compounds can stimulate rooting by preventing the decarboxylation of IAA [56]. In other cases, they can act as co-factors of IAA oxidase, stimulating its degradation [56].

### 3.3. Potential Application in Molecular Breeding

Marker-assisted selection (MAS) offers a powerful approach for accelerating the development of high-quality rice cultivars. Previous studies have successfully improved grain quality through MAS [57,58]. In our study, accessions carrying the *Rc*-H002 haplotype exhibited superior antioxidant properties and could serve as ideal donors in breeding programs. The development of functional markers for *Rc*-H002 and other candidate loci will facilitate the precise selection of favorable alleles and enable allele pyramiding strategies.

Although increasing the phenolic content of rice can enhance its nutritional value, potential negative impacts on grain yield, milling quality, or palatability must be carefully considered—particularly in terms of how phenolic compounds influence consumer acceptance of the final product. Research has shown that ferulic acid and p-coumaric acid play key roles in rice. Through thermal decarboxylation during cooking, these compounds generate volatile phenolics such as 2-methoxy-4-vinylphenol, which imparts a distinctive clove-like aroma to cooked rice [59]. However, 4-vinylphenol—a compound primarily formed from the thermal degradation of p-coumaric acid—produces an unpleasant, bran-like odor [60]. Furthermore, free phenolic acids are thought to accelerate lipid oxidation, leading to the formation of carbonyl compounds that contribute to off-flavors in cooked rice [61]. Therefore, future breeding efforts should aim to balance health benefits with agronomic performance and sensory quality to ensure the development of rice varieties that are both nutritionally rich and sensorially acceptable.

## 4. Materials and Methods

### 4.1. Materials

A total of 84 Shanlan rice accessions, including white, red, and black rice, were selected from Hainan Province (Table S1; Figure S1). All the rice accessions were cultivated and harvested at an experimental farm of Zhejiang University in Sanya, China (18.3085° N, 109.3928° E) in 2022. Following air-drying, samples were stored at room temperature for two and a half months. Rice grains were dehulled (Type THU, Satake Co., Tokyo, Japan) and ground to flour (Cyclone Sample Mill, UDY Corporation, Fort Collins, CO, USA) to pass through a 100-mesh sieve.

### 4.2. Chemicals

2,2-diphenyl-1-picrylhydrazyl(DPPH),2,2-azino-bis (3-ethylbenzothiazoline-6-sulfonic acid (ABTS), Folin–Ciocalteu reagent, Trolox, and phenolic acid standards were purchased from Sigma-Aldrich Chemical Co. (St. Louis, MO, USA). HPLC-grade methanol was purchased from Merck (Darmstadt, Germany). HPLC-grade acetic acid was purchased from Macklin (Shanghai, China). Other chemicals were purchased from Sinopharm Chemical Reagent Co., Ltd. (Shanghai, China).

### 4.3. Extraction of Free Phenolic Acids and Insoluble Bound Phenolic Acids

The extraction of free phenolic acids was modified from the method reported by Pang et al. [29]. Briefly, 0.5 g of whole-grain flour was extracted twice with 5 mL of 80% methanol. After shaking at 250 rpm for 1 h using an oscillator (Multi-speed oscillator HY-8, Changzhou Guohua Electric Appliance Co., Ltd., Changzhou City, Jiangsu Province, China), the mixture was centrifuged at 3000 rpm for 10 min. The supernatants of each duplicate extraction were collected and combined together and then stored at −20 °C until use. Duplicate extractions were performed for each sample.

The extraction method of bound phenolic acids refers to Shao et al. [14]. The solid residue after the extraction of soluble phenolics was washed with distilled water and then digested with 4 M NaOH (20 mL) at room temperature on a shaker (Multi-speed oscillator HY-8, Changzhou Guohua Electric Appliance Co., Ltd., Changzhou City, Jiangsu Province, China) for 2 h. The resultant mixture was adjusted to a pH between 1.5 and 2.0 with 6 N HCl and then extracted with 40 mL of ethyl acetate three times (20, 10, and 10 mL). The supernatant was collected and combined into an Erlenmeyer flask and then evaporated to dryness at 37 °C under vacuum using a rotary evaporator and water bath (RE-2000A, Ya Rong Biochemistry Instrument Factory, Shanghai, China). The dried residue was redissolved in 2 mL of 50% methanol and stored at −20 °C. Prior to HPLC analysis, it was filtered through a 0.45-μm syringe filter.

### 4.4. Determination of Phenolic Acid Content and Flavonoid Content

The total phenolic content (TPC) was determined using the Folin–Ciocalteu colorimetric method, with slight modifications [12]. Absorbance of each extract was measured at 725 nm using a spectrophotometer, and each measurement was performed in triplicate. Gallic acid (GA) was used as the standard for constructing the calibration curve. Results are expressed as milligrams of gallic acid equivalents per 100 g of rice (mg GAE/100 g).

The total flavonoid content (TFC) was analyzed using the soluble free phenolic extracts, following the method of Xu et al. [19]. Absorbance was measured at 510 nm, with each sample analyzed in triplicate. Catechin was employed as the standard for the calibration curve. TFC values are expressed as milligrams of catechin equivalents per 100 g of rice flour (mg CE/100 g).

### 4.5. Antioxidant Activity Determination

The radical scavenging activities against ABTS and DPPH were determined using the method described by Pang et al. [29]. Trolox was the standard sample used to construct the calibration curve. Results are expressed as micromoles of Trolox equivalent antioxidant capacity per 100 g of dry weight rice flour (μmol TE/100 g).

### 4.6. HPLC Analysis

The qualitative and quantitative analysis of the binding phenolic acid components was performed by high-performance liquid chromatography (Agilent Technologies 1260, Santa Clara, CA, USA). Separation was performed using 250 × 4.6 mm C18 columns and 5 micron particles (Agilent Eclipse Plus, Santa Clara, CA, USA). The mobile phase consists of A (0.1% acetic acid aqueous solution) and B (0.1% acetic acid methanol solution). The flow rate is 0.5 mL/min. The 35 min linear gradient is set as follows: 0–1 min, 9–25% B; 1–4 min, 25–35% B; 4–8 min, 35–45% B; 8–10 min, 45–46% B; 10–12 min, 46–47% B; 12–14 min, 47–48% B; 14–16 min, 48–48.5% B; 16–16.5 min, 48.5–49% B; 16.5–17.5 min, 49–49.1% B; 17.5–18 min, 49.1–49.5% B; 18–20 min, 49.5–50% B; 20–25 min, 50–60% B; 25–30 min, 60–9% B; 30–35 min, 9% B. The injection volume was 3 μL. The injection temperature is maintained at 35 °C. The extract was filtered through a 0.45 μm filter membrane for analysis. Phenolic acids are measured at wavelengths of 280 and 320 nm and quantified using an external calibration curve based on the retention time of the phenolic acid standard. The results were expressed as micrograms per gram dry weight of rice flour (μg/g), and all analyses were performed in duplicate. Eight phenolic acid standards were used for external calibration: gallic acid (GA), protocatechuic acid (PA), p-hydroxybenzoic acid (p-HBA), vanillic acid (VA), caffeic acid (CA), p-coumaric acid (p-CA), ferulic acid (FA), and sinapic acid (SA). The limit of quantification (LOQ) and recovery rate data are provided in Table S2. For accessions with phenolic acid content below the LOQ, the extract was concentrated and re-analyzed to enable detection.

### 4.7. Genome-Wide Association Study

The total of 3,425,822 SNPs and insertion/deletion (InDel) [24] were selected for the GWAS analysis using fixed and random model circulating probability unification (FarmCPU) in the rMVP software (version 1.1.0). The Manhattan, QQ, and SNP-density plots for GWAS were visualized using the R package CMplot (V4.3.0 https://github.com/YinLiLin/R-CMplot, accessed on 7 September 2024). Leading SNPs of each significant SNP cluster (in 200 kb) were selected to display the location of the QTLs.

### 4.8. Statistical Analysis

To ensure reliability and reproducibility, all experiments were performed with two independent biological replicates, and three technical replicates were performed per biological replicate to assess measurement precision. Duncan’s multiple comparison test of ANOVA and correlation analysis were conducted using the SPSS 25.0 software (SPSS, Inc., Chicago, IL, USA).

## 5. Conclusions

Our results demonstrate that pigmented Shanlan rice accessions exhibit significantly higher total phenolic content, flavonoid levels, and antioxidant capacity compared to white-grained accessions, highlighting the strong link between grain color and nutritional quality. HPLC analysis identified eight bound phenolic acids, with sinapic and vanillic acids being the most abundant, indicating that they are major phenolic components in Shanlan rice grains. Through GWAS, 84 loci associated with phenolic traits were detected, among which a major QTL on chromosome 7 co-localized with the *Rc* gene—a key regulator of red pericarp pigmentation. Notably, the H002 haplotype at this locus was associated with the highest antioxidant activity, making it a prime target for marker-assisted selection in rice. Additionally, a promising QTL on chromosome 11, linked to three phenolic acids, warrants further functional validation. Collectively, these findings not only elucidate the genetic basis of phenolic metabolism in Shanlan rice but also provide valuable haplotypes and candidate genomic regions for breeding.

## Figures and Tables

**Table 1:** Summary of phenolic acids and their antioxidant activity, phenotypic traits of different colored rice varieties.

Traits	White			Red			Black			Total		
	Mean	Min	Max	Mean	Min	Max	Mean	Min	Max	Mean	Min	Max
FPC ^1^	55.71 ^b^	20.99	351.83	727.66 ^a^	171.75	2310.45	340.24 ^ab^	232.73	447.75	270.47	20.99	2310.45
BPC ^1^	66.41 ^c^	22.00	108.75	106.03 ^b^	64.00	236.88	165.38 ^a^	105.88	224.88	81.03	22.00	236.88
TPC ^1^	122.12 ^b^	60.93	430.20	833.70 ^a^	249.00	2408.33	505.61 ^ab^	338.60	672.63	351.50	60.93	2408.33
TFC ^2^	19.47 ^b^	1.76	147.12	167.86 ^a^	37.88	300.32	162.52 ^a^	119.44	205.60	68.80	1.76	300.32
DPPH ^3^	341.61 ^b^	56.67	801.15	795.62 ^a^	680.39	809.63	780.50 ^a^	772.84	788.16	492.59	56.67	809.63
ABTS ^3^	274.96 ^b^	31.95	1217.84	1262.58 ^a^	529.93	1917.77	957.00 ^a^	544.81	1369.20	596.89	31.95	1917.77
GA ^4^	0.29 ^a^	0.06	1.88	0.25 ^a^	0.06	0.63	0.51 ^a^	0.10	0.92	0.28	0.06	1.88
PA ^4^	0.44 ^a^	0.04	2.88	0.34 ^a^	0.12	1.06	0.51 ^a^	0.13	0.90	0.41	0.04	2.88
p-HBA ^4^	0.33 ^a^	0.03	1.02	0.39 ^a^	0.06	3.29	0.59 ^a^	0.20	0.98	0.35	0.03	3.29
CA ^4^	0.32 ^a^	0.07	6.22	0.30 ^a^	0.08	1.96	0.36 ^a^	0.22	0.51	0.31	0.07	6.22
VA ^4^	12.41 ^a^	0.26	53.60	9.94 ^a^	1.83	17.37	15.46 ^a^	7.84	23.08	11.72	0.26	53.60
p-CA ^4^	4.35 ^a^	0.30	18.08	3.52 ^a^	0.82	6.00	5.36 ^a^	2.82	7.90	4.12	0.30	18.08
SA ^4^	59.57 ^a^	0.99	237.46	44.33 ^a^	27.03	63.24	69.29 ^a^	36.43	102.14	55.08	0.99	237.46
FA ^4^	1.45 ^a^	0.10	4.94	1.26 ^a^	0.10	2.40	1.32 ^a^	1.25	1.38	1.39	0.10	4.94

GA: gallic acid; PA: protocatechuic acid; p-HBA: p-hydroxybenzoic acid; CA: caffeic acid; VA: vanillic acid; p-CA: p-coumaric acid; SA: sinapic acid; FA: ferulic acid; ^1^: mg gallic acid equivalent/100 g; ^2^: mg catechol equivalent/100 g; ^3^: μmol Trolox equivalent/100 g; ^4^: μg/g. Different lowercase letters within the same row indicate significant differences at *p* < 0.05 according to Duncan’s multiple comparison test.

**Table 2:** Correlation analysis of phenolic acids and antioxidant activity.

	FPC	BPC	TPC	TFC	DPPH	ABTS	GA	PA	p-HBA	CA	VA	p-CA	SA
BPC	0.293 **												
TPC	0.998 **	0.354 **											
TFC	0.719 **	0.570 **	0.742 **										
DPPH	0.591 **	0.624 **	0.620 **	0.843 **									
ABTS	0.774 **	0.530 **	0.793 **	0.959 **	0.837 **								
GA	−0.065	−0.014	−0.064	0.006	0.038	−0.051							
PA	−0.159	−0.123	−0.164	−0.131	−0.093	−0.172	0.555 **						
p-HBA	−0.051	0.124	−0.041	0.091	0.119	0.052	0.394 **	0.340 **					
CA	−0.049	0.065	−0.044	0.027	0.035	−0.015	0.082	0.078	0.116				
VA	−0.046	−0.024	−0.047	−0.135	−0.224 *	−0.123	0.067	0.14	0.034	0.125			
p-CA	−0.046	−0.024	−0.047	−0.135	−0.224 *	−0.123	0.067	0.14	0.034	0.125	1.000 **		
SA	−0.089	−0.029	−0.089	−0.14	−0.243 *	−0.151	0.161	0.144	0.049	0.276 *	0.941 **	0.941 **	
FA	−0.015	0.077	−0.009	−0.092	−0.129	−0.076	0.021	0	0.075	0.411 **	0.712 **	0.712 **	0.757 **

Pearson correlation coefficients are indicated; * and ** indicate significant correlations at *p* < 0.05 and *p* < 0.01, respectively (two-tailed test).

**Table 3:** GWAS-identified loci associated with phenolic acid-related traits.

Trait	Position ^1^	Polymorphism	*p*-Value	Effect	Candidate Locus	Region	Known Gene
FPC	S4_8608872	T/C	9.28 × 10^−10^	−231.75	LOC_Os04g15870	exonic	
	S5_27501155	G/A	1.20 × 10^−11^	206.42	LOC_Os05g47960 (dist = 1569)	upstream	
	S6_9901517	C/G	1.21 × 10^−7^	−103.99	LOC_Os06g17070 (dist = 6349) LOC_Os06g17080 (dist = 2583)	intergenic	
	S7_6068071	A/AACGCGAAAAGTCGG	4.13 × 10^−27^	159.47	LOC_Os07g11020	exonic	*Rc*
	S7_16138785	A/G	6.91 × 10^−14^	151.12	LOC_Os07g27630 (dist = 3320) LOC_Os07g27650 (dist = 6394)	intergenic	
	S8_15935332	G/A	7.28 × 10^−8^	139.96	LOC_Os08g26200 (dist = 1553) LOC_Os08g26190 (dist = 1105)	upstream downstream	
	S12_6284651	G/A	2.82 × 10^−14^	217.92	LOC_Os12g11590	exonic	
BPC	S2_2531849	G/A	4.88 × 10^−8^	−5.49	LOC_Os02g05260	exonic	
	S3_27983323	T/C	3.33 × 10^−8^	14.99	LOC_Os03g49126 (dist = 526) LOC_Os03g49120 (dist = 1360)	upstream downstream	
	S5_5790070	ACAGGTCCTC/A	8.40 × 10^−8^	−13.37	LOC_Os05g10620, LOC_Os05g10625 (dist = 138) LOC_Os05g10630 (dist = 138)	upstream downstream	
	S6_574213	T/C	3.08 × 10^−17^	−96.32	LOC_Os06g02010	exonic	
	S6_14781772	G/A	5.39 × 10^−9^	11.80	LOC_Os06g25270	exonic	
	S7_6170573	G/A	2.41 × 10^−16^	−11.67	LOC_Os07g11200	exonic	
	S8_16402811	C/T	7.12 × 10^−8^	7.81	LOC_Os08g26890 (dist = 12845) LOC_Os08g26900 (dist = 2170)	intergenic	
TPC	S3_7796717	T/C	1.61 × 10^−30^	734.65	LOC_Os03g14320	exonic	
	S4_5262699	ATTGCAAGGAGTCG/A	3.66 × 10^−8^	−125.76	LOC_Os04g09810 (dist = 957)	downstream	
	S4_8505441	G/C	1.41 × 10^−10^	120.20	LOC_Os04g15640	exonic	
	S4_21608571	C/T	1.81 × 10^−24^	300.67	LOC_Os04g35510 (dist = 802)	downstream	
	S7_6068071	A/AACGCGAAAAGTCGG	4.29 × 10^−33^	187.80	LOC_Os07g11020	exonic	*Rc*
	S8_3232230	A/G	5.86 × 10^−17^	556.67	LOC_Os08g05940 (dist = 4005) LOC_Os08g05950 (dist = 7398)	intergenic	*pds1*
	S8_14227902	G/A	8.32 × 10^−10^	−149.50	LOC_Os08g23500 (dist = 1942)	upstream	
	S9_6298533	C/T	2.64 × 10^−24^	400.40	LOC_Os09g11330 (dist = 2581) LOC_Os09g11350 (dist = 2388)	intergenic	
	S11_24663643	T/G	4.35 × 10^−57^	892.65	LOC_Os11g41160 (dist = 4315) LOC_Os11g41170 (dist = 7324)	intergenic	
TFC	S1_890709	A/G	7.51 × 10^−12^	−18.73	LOC_Os01g02650 (dist = 1386)	upstream	
	S1_18037853	G/T	2.95 × 10^−9^	17.76	LOC_Os01g32864 (dist = 1287)	upstream	
	S2_33906691	G/A	5.83 × 10^−8^	−12.47	LOC_Os02g55350	intronic	
	S4_23251954	T/C	1.38 × 10^−10^	−19.71	LOC_Os04g39110 (dist = 8752) LOC_Os04g39120 (dist = 17585)	intergenic	*OsGASR2*
	S5_29897937	G/A	7.87 × 10^−21^	46.76	LOC_Os05g52070	exonic	
	S7_6068071	A/AACGCGAAAAGTCGG	1.51 × 10^−50^	66.24	LOC_Os07g11020	exonic	*Rc*
	S10_11143309	T/A	2.28 × 10^−8^	12.83	LOC_Os10g21700 (dist = 14656) LOC_Os10g21720 (dist = 3752)	intergenic	
	S11_18026280	T/C	2.69 × 10^−22^	40.23	LOC_Os11g30990	exonic	
	S12_9386926	T/G	1.38 × 10^−7^	−16.13	LOC_Os12g16400	intronic	
	S12_18137641	G/A	9.03 × 10^−9^	20.53	LOC_Os12g30214	exonic	
DPPH	S1_2542760	C/T	6.65 × 10^−14^	−114.38	LOC_Os01g05370 LOC_Os01g05380 (dist = 28)	upstream	
	S1_3917148	C/A	1.73 × 10^−10^	50.52	LOC_Os01g08090 (dist = 770)	upstream	
	S1_16259756	G/C	6.99 × 10^−12^	−48.66	LOC_Os01g29040 LOC_Os01g29050 (dist = 485)	upstream	
	S5_5680332	C/T	3.12 × 10^−10^	−66.34	LOC_Os05g10430	intronic	
	S6_11653435	C/T	1.24 × 10^−13^	−87.88	LOC_Os06g20290 (dist = 387)	downstream	
	S7_6068071	A/AACGCGAAAAGTCGG	2.95 × 10^−51^	222.93	LOC_Os07g11020	exonic	*Rc*
	S8_4407898	C/A,G	6.67 × 10^−34^	215.49	LOC_Os08g07850	intronic	
	S11_28201425	C/G	7.93 × 10^−13^	−67.71	LOC_Os11g46920	exonic	
ABTS	S1_27531822	T/C	4.96 × 10^−9^	159.97	LOC_Os01g49920	exonic	
	S2_27557707	C/T	1.64 × 10^−7^	117.95	LOC_Os02g45344 (dist = 1496)	upstream	
	S4_23223209	A/G	2.63 × 10^−15^	−161.85	LOC_Os04g39070 (dist = 1291)	upstream	
	S5_24882400	AGAGG/A	6.41 × 10^−10^	−120.26	LOC_Os05g42428 (dist = 4750) LOC_Os05g42436 (dist = 26926)	intergenic	
	S5_29897937	G/A	9.58 × 10^−17^	290.46	LOC_Os05g52070	exonic	
	S7_6068071	A/AACGCGAAAAGTCGG	2.07 × 10^−40^	444.09	LOC_Os07g11020	exonic	*Rc*
	S11_18026280	T/C	7.95 × 10^−15^	183.84	LOC_Os11g30990	exonic	
	S11_20846878	G/A	1.41 × 10^−8^	143.26	LOC_Os11g35560	intronic	
	S12_23293153	G/A	5.02 × 10^−11^	121.36	LOC_Os12g37900 (dist = 309) LOC_Os12g37890 LOC_Os12g37910 (dist = 309)	upstream downstream	
GA	S1_26909462	A/AC	6.68 × 10^−11^	−0.14	LOC_Os01g47090 (dist = 7818) LOC_Os01g47100 (dist = 3135)	intergenic	
	S2_34727049	A/G	1.20 × 10^−10^	0.14	LOC_Os02g56640	exonic	
	S3_20453194	A/T	1.46 × 10^−14^	0.26	LOC_Os03g36860	exonic	
	S4_4113997	A/G	5.07 × 10^−8^	0.14	LOC_Os04g07730	exonic	
	S5_3991938	A/C	1.69 × 10^−15^	0.45	LOC_Os05g07470 (dist = 8338) LOC_Os05g07480 (dist = 6747)	intergenic	
	S8_166588	A/G	5.41 × 10^−10^	−0.14	LOC_Os08g01260 (dist = 1372)	upstream	*OsVQ32*
	S11_26976494	C/T	4.98 × 10^−13^	0.22	LOC_Os11g44600	exonic	
PA	S1_23469338	A/C	2.26 × 10^−14^	0.36	LOC_Os01g41450 (dist = 4153) LOC_Os01g41460 (dist = 2881)	intergenic	
	S2_19213738	A/C	1.06 × 10^−9^	−0.23	LOC_Os02g32480 (dist = 916)	downstream	
	S2_30352381	GC/G	2.70 × 10^−10^	−0.40	LOC_Os02g49670 (dist = 317)	downstream	
	S5_27988411	T/C	2.60 × 10^−13^	−1.15	LOC_Os05g48830	exonic	
	S8_10428981	C/T	2.21 × 10^−11^	−0.25	LOC_Os08g17030	exonic	
	S10_4396593	A/T	1.38 × 10^−7^	0.14	LOC_Os10g08140	intronic	
p-HBA	S1_24264360	T/C	8.87 × 10^−14^	−0.59	LOC_Os01g42660(dist = 156)	upstream	
	S1_28671305	A/C,T	5.15 × 10^−44^	1.48	LOC_Os01g49920	exonic	
	S1_41031628	C/T	8.14 × 10^−17^	0.34	LOC_Os01g70890 LOC_Os01g70900 (dist = 147)	upstream	*OsNF-* *YB6* *OsHAP3G*
	S5_9091371	C/T	1.32 × 10^−9^	0.16	LOC_Os05g16080 (dist = 5811) LOC_Os05g16090 (dist = 2759)	intergenic	
	S6_2827762	T/C	1.13 × 10^−7^	−0.07	LOC_Os06g06115 (dist = 681)	upstream	
	S9_15098602	TTCGTC/N,TGTCGTC	1.92 × 10^−7^	−0.07	LOC_Os09g25200 (dist = 758)	downstream	
	S11_8262262	G/A	4.46 × 10^−10^	0.15	LOC_Os11g14650	intronic	
	S11_11150178	G/A	9.82 × 10^−8^	0.13	LOC_Os11g19380 (dist = 551)	upstream	
p-CA /VA	S2_551933	G/A	1.43 × 10^−16^	2.81	LOC_Os02g01990 (dist = 751)	upstream	*DHD4*
	S4_6167066	C/T	3.15 × 10^−12^	1.25	LOC_Os04g11290	exonic	
	S4_13289104	C/T	1.29 × 10^−7^	−1.18	LOC_Os04g23280	intronic	
	S5_16026627	G/A	2.18 × 10^−12^	2.29	LOC_Os05g27540	exonic	
	S7_9790670	ACGATGGTAAATCG/A	2.39 × 10^−9^	1.70	LOC_Os07g16690 (dist = 215)	upstream	
	S11_21594350	C/T	3.02 × 10^−8^	1.72	LOC_Os11g36580 (dist = 704)	upstream	
SA	S1_29034197	C/T	1.80 × 10^−8^	13.55	LOC_Os01g50550 (dist = 784) LOC_Os01g50560 (dist = 1076)	upstream downstream	
	S5_15307396	T/C	5.67 × 10^−8^	−10.21	LOC_Os05g26310 LOC_Os05g26320 (dist = 1876)	downstream	
	S7_13188853	G/A	2.75 × 10^−8^	−22.77	LOC_Os07g23400	exonic	
	S7_21496930	G/A	1.01 × 10^−13^	32.74	LOC_Os07g35920	intronic	
	S11_21594350	C/T	6.84 × 10^−13^	32.01	LOC_Os11g36580 (dist = 704)	upstream	
	S12_26390982	A/G	5.30 × 10^−22^	40.70	LOC_Os12g42480 (dist = 626)	upstream	
FA	S1_38008134	C/CTATTT	3.08 × 10^−19^	−0.72	LOC_Os01g65480 (dist = 1492) LOC_Os01g65470 (dist = 1494)	upstream downstream	
	S3_7652662	C/A	4.54 × 10^−8^	0.19	LOC_Os03g14080 (dist = 2103) LOC_Os03g14090 (dist = 3520)	intergenic	*OsAUX2*
	S3_14331413	A/G	1.57 × 10^−9^	0.26	LOC_Os03g25100	exonic	
	S5_7616250	A/T	1.33 × 10^−24^	1.30	LOC_Os05g13740 (dist = 89)	downstream	
	S6_5891020	T/G	9.46 × 10^−10^	−0.42	LOC_Os06g11220 LOC_Os06g11230 (dist = 51)	downstream	
	S8_16274914	A/G	1.98 × 10^−15^	−0.40	LOC_Os08g26770 (dist = 44) LOC_Os08g26760 (dist = 325)	upstream downstream	
	S8_17348215	G/A	5.60 × 10^−15^	0.59	LOC_Os08g28410	intronic	
	S9_4720926	A/AT	1.55 × 10^−15^	−0.57	LOC_Os09g08900 (dist = 66)	downstream	

^1^ The letter S indicates a SNP or insertion/deletion, the first number after S indicates the chromosome, and the second number indicates its physical position.

## Data Availability

The original contributions presented in the study are included in the article/Supplementary Materials. Further inquiries can be directed to the corresponding author.

## Supplementary Materials

The following supporting information can be downloaded at: https://www.mdpi.com/article/10.3390/ijms26199800/s1.
