# Supplementary material for: Genotypic Variation and Genetic Control of Phenolic Compounds and Antioxidant Activity in Shanlan Upland Rice Landrace
Source: Int J Mol Sci. 2025 Oct 8;26(19):9800. doi: 10.3390/ijms26199800 (PMC12525001; doi:10.3390/ijms26199800)
Supplement: Supplementary file 1 [file ijms-26-09800-s001.zip › ijms-3888193-supplementary.pdf]

Table S1 The 84 rice accessions of Shanlan landrace rice

Code	Name	Sub-population	Color
SL01	Menjiading3	XI	white
SL02	Baikezhan4	XI	white
SL03	Shenshuilian	XI	white
SL04	menjiading2	XI	white
SL05	hualinuo2	GJ2	white
SL06	menjiaqin	GJ2	white
SL07	menjianing	GJ2	white
SL08	miaomumai2	XI	white
SL09	zhishouwan1	GJ2	red
SL10	zhishouwan2	GJ2	white
SL11	menmiaonong1	GJ2	white
SL12	menmiaonong2	GJ2	white
SL13	mennuo1	GJ2	white
SL14	huanggu1	GJ2	white
SL15	huanggu2	GJ2	red
SL16	zhuangannuo	GJ2	white
SL17	shanlannuo1	GJ2	white
SL18	shanlannuo2	GJ2	red
SL19	shanlannuo3	GJ2	white
SL20	hongkezidaoyan	XI	red
SL21	guangtounuo	GJ2	white
SL22	shanlan2	XI	white
SL23	pole2	XI	red
SL24	menjiaoyan	GJ2	white
SL25	menjiade2	XI	white
SL26	baisilianggan	GJ2	white
SL27	menjiaoyan1	GJ2	white
SL28	shanlannuo3	GJ1	red
SL29	moyiqiu	GJ2	white
SL30	heisidi1	GJ2	white
SL31	pozhan1	XI	red
SL32	pozhan3	XI	red
SL33	daohuangnuo	XI	white
SL34	heimaonuo	GJ2	white

SL35	zhongping1	GJ2	white
SL36	zhongping2	GJ2	white
SL37	zhongping3	GJ2	red
SL38	zhongping4	GJ2	white
SL39	zhongpingnian1	XI	white
SL40	zhongpingnian2	GJ2	white
SL41	menkao2	XI	white
SL42	wuke3	XI	red
SL43	wuke4	XI	red
SL44	menjiafei1	XI	red
SL45	shanlan3	XI	red
SL46	huangweishan2	XI	red
SL47	menjiamei1	XI	white
SL48	menjiamei2	XI	red
SL49	jialai1	GJ2	white
SL50	kajialai2	GJ2	white
SL51	gugu1	XI	red
SL52	gugu3	GJ2	white
SL53	huangnuo3	XI	white
SL54	heidao1	XI	white
SL55	wanningnuo1	XI	white
SL56	shengsuanshanlan	GJ2	red
SL57	menli	XI	red
SL58	shanlannuo3	GJ2	white
SL59	lvdao	GJ1	white
SL60	shanlannuo3(hongmang)	XI	white
SL61	menjiati1	GJ1	white
SL62	menjiati4	GJ1	red
SL63	pohe	XI	white
SL64	shanlanzhumudao	GJ2	red
SL65	lizhishanlan1	XI	white
SL66	wuyuehong	XI	red
SL67	fanjia	GJ2	white
SL68	heimaozhan	GJ2	white
SL69	shanlan1	GJ2	white
SL70	shanlanuo2	GJ2	white
SL71	heinuo	XI	black

SL72	jinsi	XI	white
SL73	zhaonong	XI	white
SL74	shanlannuo2	XI	red
SL75	shanlannuo3	XI	white
SL76	shanlannuo4	GJ2	white
SL77	guangtounuo	XI	white
SL78	baishanlan	XI	white
SL79	shanlanbainuo	XI	red
SL80	shuangkehongmi	GJ2	red
SL81	shanlanhongmi	XI	red
SL82	shanlanheimi	XI	white
SL83	shanlandao1	XI	black
SL84	shanlandao2	GJ1	red

Table S2. Regression equations, limits of detection (LOD), limits of quantification (LOQ), and recovery rates for analysis of the phenolic acids in rice.

Phenolic acids	Wavelength （nm）	Regression equations	Correlation coefficient (R)	Linear range (μg/mL)	Limits of detection (μg/mL)	Limits of quantification (μg/mL)	Recovery (%)
Gallic acid	280	y=17.786x-0.3636	1.0000	0.1-16	0.02	0.06	96.34
Protocatechuic acid	280	y=9.0165x-1.2751	0.9980	0.1-16	0.02	0.06	97.23
p-Hydroxybenzoic acid	280	y=10.24x-0.295	0.9980	0.1-16	0.02	0.05	103.89
Vanillic acid	280	y=10.659x+0.1567	0.9990	0.1-20	0.02	0.06	97.68
Caffeic acid	320	y=19.341x+0.280	0.9990	0.1-16	0.02	0.06	98.26
p-Coumaric acid	320	y=30.752+6.513	1.0000	0.1-16	0.02	0.06	95.12
Ferulic acid	320	y=17.943x+5.5639	1.0000	0.1-16	0.002	0.005	95.61
Sinapic acid	320	y=5.4418x+0.5864	0.9997	0.1-80	0.03	0.08	106.14

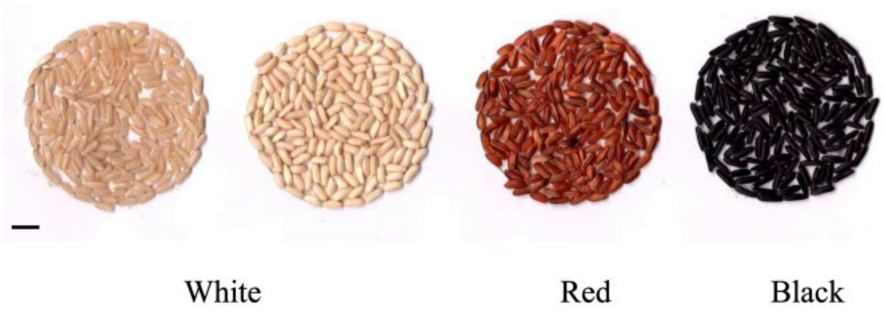

Figure S1. Classification of Shanlan rice accessions based on seed color: White, Red, and Black. Scale bar = 1 cm.
